# Preventing Colorectal Cancer: Pathway to Achieving an 80% Screening Goal in the United States: Overview and Proceedings of a Population Health Advisory Board

**DOI:** 10.1089/pop.2020.0076

**Published:** 2021-04-09

**Authors:** David B. Nash, Raymond J. Fabius, Alexis Skoufalos

**Affiliations:** ^1^ Jefferson College of Population Health, Philadelphia, Pennsylvania, USA.; ^2^ AB3 Health LLC, Philadelphia, Pennsylvania, USA.

**Keywords:** colorectal cancer, mt-sDNA test, patient navigation program, preventive screening guidelines, patient and provider adherence, role for employers

## Editorial

What sets colorectal cancer (CRC) apart from other forms of cancer as a serious population health issue? The succinct but profound answer is that it is the “most preventable yet least prevented” form of cancer.^1^

Because CRC usually does not cause symptoms until the disease is well advanced, timely preventive screening is generally understood to be vitally important. But despite the availability of multiple modalities, ranging from at-home tests to colonoscopy procedures, adherence to CRC screening guidelines remains alarmingly low in the United States. For example, one study of a continuously insured population showed a mere 64% adherence to the US Preventive Services Task Force (USPSTF) recommendations over a 10-year period. Of the individuals who were tested, more than 99% were screened via colonoscopy.^2^

The National Colorectal Cancer Roundtable (NCCRT) has set a goal of achieving screening rates of 80% in every community across the United States.^3^ Although ambitious, this goal is realistically attainable given the number of nationally recommended screening tests that range from easily obtained stool samples to more invasive structural examinations.

In 2014, health technology innovator Exact Sciences, Corp. (Exact Sciences) introduced a noninvasive CRC screening test using principles of biology, chemistry, and molecular biology. This powerful combination of technologies is packaged into a convenient and highly effective CRC screening test. The multi-target stool DNA (mt-sDNA or Cologuard^®^) test employs a multi-marker approach to detect hemoglobin and altered DNA (mutations and methylations) that is a distinguishing feature of the company's technology platform.

We view this new technology as a potential game changer in closing the CRC screening gap. The accruing evidence suggests that the mt-sDNA test is a cost-effective alternative to colonoscopy and other stool-based tests (fecal immunochemical test [FIT], fecal occult blood test [FOBT]) for a significant portion of the population.^4,5^ That said, a compelling case can be made for the test as a beneficial, population health-based intervention – particularly in a value-based payment setting. This is good news but, as in most cases, the devil is in the details.

Although it is difficult to change established practice patterns, prior generations of clinicians were convinced to order colonoscopy when endoscopy became widely available rather than performing digital rectal examinations. Today's clinicians routinely order colonoscopy as a first-line CRC screening test; the next step may be convincing them to consider the mt-sDNA test as an option for patients at average risk for CRC because of its demonstrated clinical value, 3-year test interval, and appeal to patients.

As with most preventive health behaviors, patient adherence to CRC screening recommendations is a challenge. We believe that employers are a potentially influential sector and a largely untapped resource in the effort to increase CRC screening rates. Communication about, and promotion of, preventive screening by employers goes well beyond improving the health status of employees. It adds value by effectively reducing the direct and indirect costs of the disease.

In collaboration with Exact Sciences, the Jefferson College of Population Health (JCPH) organized and convened a 1-day advisory board meeting of health professionals to discuss the value proposition of mt-sDNA for CRC screening in terms of clinical quality, risk management, finances, and population health management.

## Overview/Background

Highly prevalent and disturbingly insidious, colorectal cancer (CRC) imposes a heavy toll on the health of the US population. It is the second leading cause of cancer mortality and is expected to cause about 53,200 deaths by the end of 2020.^6^ With nearly 150,000 new cases diagnosed per year, health care costs are estimated to approach $14 billion for the 10-year period ending in 2020.^2,6^

**FIG. 1.**
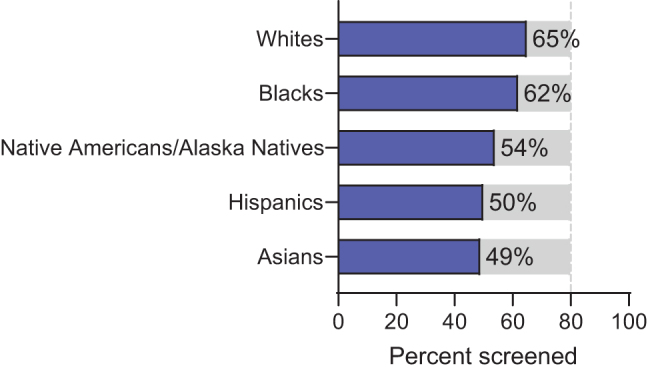
Colorectal screening rates (%) by ethnic group (2015). Adapted with permission Source: American Cancer Society. Colorectal Facts & Figures 2017–2019. https://www.cancer.org/content/dam/cancer-org/research/cancer-facts-and-statistics/colorectal-cancer-facts-and-figures/colorectal-cancer-facts-and-figures-2017-2019.pdf Accessed March 25, 2020.

Overall, the lifetime risk of developing CRC is about 4.49% for men (1 in 22) and 4.15% in women (1 in 24). Risk factors include personal and family history, physical inactivity and obesity, nutritional status, smoking, and race. Black Americans have the highest rates of nonhereditary CRC, and CRC is the leading cause of death in this population, particularly among black women^7^ (Figure 1).

As a result of increased screening and improved treatments, the overall death rate from CRC has decreased gradually over the last decade.^8^ However, deaths from CRC among people younger than age 55 have increased 1% each year from 2007 through 2016.^6^ Consequently, the American Cancer Society recently lowered the recommended age for beginning preventive screening from 50 to 45.^9^

**FIG. 2.**
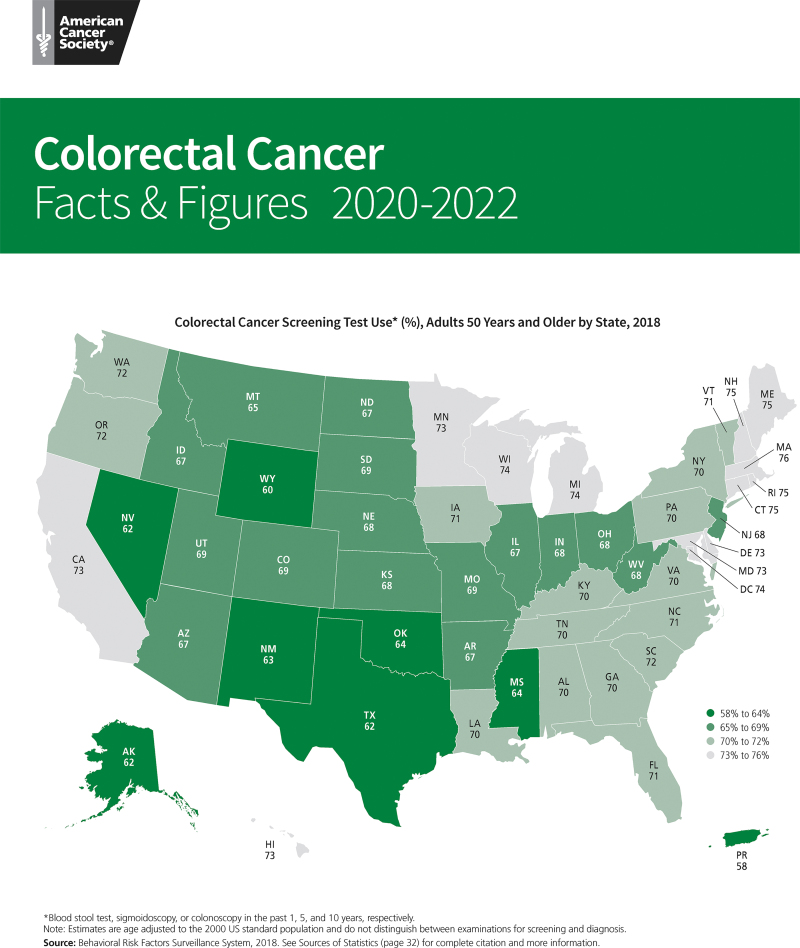
US colorectal cancer screening test use (%) in adults 50 years and older by state (2018). Reproduced with permission from the American Cancer Society. Colorectal cancer facts and figures, 2020–2022. https://www.cancer.org/content/dam/cancer-org/research/cancer-facts-and-statistics/colorectal-cancer-facts-and-figures/colorectal-cancer-facts-and-figures-2020-2022.pdf Accessed March 25, 2020.

According to a 2016 retrospective claims analysis of a continuously insured population, only 64% of the target population adhered to USPSTF recommendations for CRC screening.^10^ In fact, screening rates remain below the nationwide target of 80% regardless of state, age, or ethnicity^11^ (Figure 2).

As in many cancerous conditions, early detection of CRC is critical to favorable outcomes. If diagnosed at Stage I, 9 out of every 10 patients survive at least 5 years.^11^ However, statistics show that only 39% of CRCs are diagnosed at Stage I or II.^11^ Prospects of survival diminish substantially for patients diagnosed at later stages. Statistics reveal that, of the 61% of patients diagnosed at Stages III or IV, the 5-year survival rate drops to 1 out of every 10 patients.^11^

Low screening rates can be attributed in part to reported barriers at the patient, provider, and system levels.^12^ Patient-reported barriers include fear of a test, unpleasant prep, lack of knowledge, physical discomfort associated with a test, cost or lack of insurance, fear of learning the results, and inconvenience.^12^ System-level barriers perceived by providers include lack of an effective strategy (eg, reminders) to promote cancer screening and lack of support staff to utilize such resources.^13^

Current preventive testing guidelines (eg, American Cancer Society, USPSTF, National Comprehensive Cancer Network) and quality measures (eg, Healthcare Effectiveness Data and Information Set [HEDIS]) specify a variety of cancer screening options that may be presented to patients at average risk for CRC. Stool-based tests and recommended frequencies include FOBT every year, FIT every year, and mt-sDNA every 3 years.^14^ Structural examinations and recommended examination intervals include flexible sigmoidoscopy every 5 years, computerized tomography colonography every 5 years, and colonoscopy every 10 years. To date, no specific test is recommended for CRC screening, and there are no head-to-head studies demonstrating the superiority of one over the others in terms of effectiveness.^15^ However, the recommendation for CRC screening includes offering patients the opportunity to select either a structural (visual) examination or a high-sensitivity stool-based test, depending on patient preference and test availability.^9^

Exact Sciences, an innovative health technology company, designed the mt-sDNA test to improve the accuracy of noninvasive CRC screening, facilitate provider and patient adherence to screening guidelines, and reduce the total cost of CRC care. Leveraging principles of biology, chemistry, and molecular biology, the mt-sDNA test packages several technologies in a single, highly effective precancer and cancer screening test. The test's combined 11 molecular biomarkers generate a “positive” or “negative” CRC screening result. Positive mt-sDNA test results should be followed up by a diagnostic colonoscopy, and individuals with negative results who remain at average risk for CRC should be rescreened for CRC in 3 years.^9^

A 10,000-patient study showed that the mt-sDNA test is highly sensitive in comparison with FIT (92% sensitivity vs. 74% with FIT). The specificity of mt-sDNA is 87% versus 95% for FIT; however, measured over a 3-year period (3 annual FIT tests, 1 mt-sDNA test), specificities are similar.^15^

Understanding the total cost of care associated with screening tests is an important element in value-based care. Given the substantial variation in recommended CRC testing modalities, direct cost comparisons are challenging at best. Even for a particular modality, screening costs may vary considerably.^16,17^ For instance, costs associated with negative screening colonoscopy procedures in the United States may range from $700–$2000 depending on the type of sedation, setting, health system, and insurance coverage. However, it is generally accepted that the total cost of any CRC screening test (eg, FIT, mt-sDNA) should include direct nonmedical costs, and indirect administrative, programmatic, and navigation costs.^18,19^

The direct and indirect cost burden of CRC is substantial for patients, families/caregivers, employers, and public and commercial payers. First-year treatment costs alone are staggering; $57,901 for patients diagnosed in Stages I or II and $108,599 for patients diagnosed in Stage IV.^20^ With more than 60% of patients diagnosed in Stages III and IV, there is a clear opportunity to improve outcomes and lower costs through screening and early detection.

In addition to being a lower cost alternative to colonoscopy for a substantial portion of the population, the mt-sDNA test is supported by an embedded nationwide patient navigation program that promotes patient adherence, addresses questions related to completing the test, and manages inbound calls from health care providers to the central laboratory. The program includes patient outreach and reminders during the first month after the test order is received. Telephonic support is available on a 24/7/365 basis for patients and health care providers in more than 240 languages.

The company aims to partner with payers and employers (who purchase health insurance plans for employees) in its effort to increase early detection of CRC. According to the Kaiser Family Foundation, employer-sponsored health insurance plans cover more than 50% of the US non-elderly population, and national data reveal CRC screening rates of less than 50% for the population of individuals aged 50–54.^21^ Although nearly 80% of employers offer wellness programs, preventive screening for CRC and other diseases appears to be underutilized. For this reason, the company seeks to create value and unique offerings that expand access to preventive services, maximize workforce health status and productivity, and reduce overall costs of CRC.

## Proceedings

In collaboration with Exact Sciences, JCPH organized and convened a 1-day advisory meeting of key stakeholders to discuss the value proposition of using mt-SDNA for CRC screening in terms of clinical quality, risk management, finances, and population management. The September 13, 2019 meeting provided an opportunity for the Exact Sciences team to connect with a range of health professionals with expertise in population-based clinical service, quality improvement, hospital and integrated delivery system risk management, and health policy.

**POPULATION HEALTH ADVISORY BOARD MEMBERS**

***Rajesh Davda, MD, MBA****, National Medical Director, Network Performance Evaluation and Improvement, Cigna Healthcare, Hartford, CT*

***Raymond J. Fabius, MD****, Principal, AB3 Health LLC, Philadelphia, PA*

***Kimberly Hutton, MD***, *Chief Medical Officer, Care ATC, Tulsa, OK*

***Michael D. Lappi, DO, MPH****, Chief Health Officer, Corning Inc., New York, NY*

***Ginger Miller, OTR/L, CLT, CEAS***, *Health Promotion Manager, Utz Quality Foods LLC, Hanover, PA*

***Ronald E. Myers, DSW, PhD***, *Professor and Director, Division of Population Health Science and Center for Health Decisions, Thomas Jefferson University, Philadelphia, PA*

***Steven Peskin, MD, MBA, FACP****, Executive Medical Director, Population Health and Transformation, Horizon Blue Cross/Blue Shield of New Jersey, Newark, NJ*

***Angela Sherwin, MPH,*** *President, Population Health MSO, Steward Health Care Network, Providence RI*

***Mark D. Smith, MD, MBA***, *Clinical Professor of Medicine, University of California, San Francisco, CA*

Moderated by ***David B. Nash, MD, MBA,*** (*Founding Dean Emeritus, JCPH*), the meeting was structured to facilitate discussion around the following questions:

1. How can the value of mt-SDNA be demonstrated to payers, providers, and employers via (1) population health strategies and initiatives and (2) a value-based payment paradigm?
2. How can technology be leveraged more effectively to engage consumers?
3. How can Exact Sciences make a greater impact on helping all stakeholders meet the NCCRT US goal of 80% screening of eligible persons in every community?

The goal of the meeting was to learn more about ways in which the company might extend and amplify its work and support consumers to choose the CRC screening test that is best for them.

## Access, Outcomes and Population Health: Exact Sciences Perspective

***Laura T. Housman, MPH, MBA*** *(Head of Access, Outcomes, and Population Health, Exact Sciences)* presented a high-level overview of Exact Sciences. By partnering with health care providers, payers, patients, and advocacy groups to help eradicate colon cancer, the company envisions playing a meaningful role in winning the war on cancer through early detection.

The advantages of the company's product include:

1) The mt-sDNA test includes 11 molecular biomarkers (including hemoglobin) that are analyzed to provide a single “positive” or “negative” result; a “positive” result indicates the need for a follow-up colonoscopy.
2) A state-of-the-art central laboratory that performs proprietary DNA chemistry.
3) A flexible automated platform.
4) Regulatory approval (parallel review Food and Drug Administration approval and Centers for Medicare & Medicaid Services national coverage decision).
5) Commercial scale operations.

Substantial investments the company has made in access, outcomes, and population health have resulted in greater involvement of employers as both payers and workplace health care innovators. Efforts also have spurred the development of patient/consumer-initiated physician ordered testing, retail outlet pathways to reach patients/consumers (eg, CVS, retail stores), and a rise in population health-based research and programs and health economics and outcomes research (eg, addressing patient care gaps, reducing disparities).

The company's strategic focus is on creating value for customers across multiple core areas related to cancer diagnostics and treatment. For instance, via a contract with the state of California, mt-sDNA is offered to Medicaid fee-for-service enrollees across the system. Currently, more than 94% of patients using mt-sDNA report having no out-of-pocket costs.

Advisor insights were requested regarding (*a*) how programmatic population health approaches improve access to wellness and prevention offerings, and (*b*) how to improve engagement and adoption of mt-sDNA as a first-line screening choice for individuals at average risk for CRC.

### Key discussion points

- Experts advised focusing on outcomes reported by recognized benchmark employers to determine whether wellness programs are having the desired effect in terms of improving preventive screening rates.
- With respect to the impact of incentives on engagement, experts advised pursuing large health systems with a systems strategy in which economic incentives are aligned with clinical incentives. Collaborative work relationships make things happen.
- Regarding the impact of cost sharing on uptake of preventive services, experts advised working with state health programs (eg, New Jersey's preventive services program).

## Population Health Strategies and Opportunities for CRC Screening

**FIG. 3.**
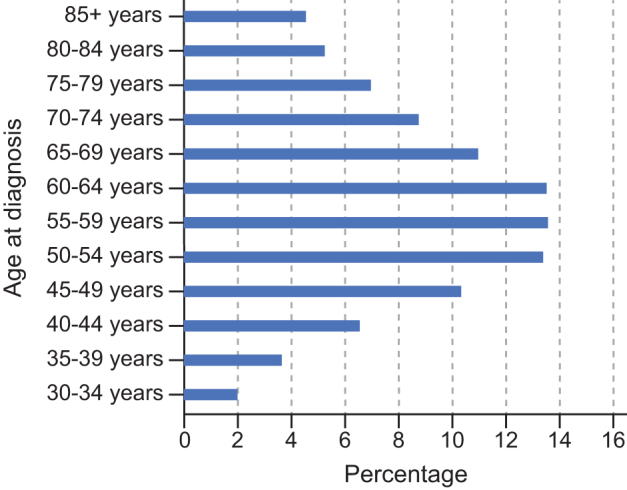
Distribution of colorectal cancer screening burden by age at diagnosis (2010–2014): person-years of life lost because of colorectal cancer by age at diagnosis among patients followed for 20 years after diagnosis. EHR, electronic health record. Adapted with permission from John Wiley and Sons. Wolf AMD, Fontham ETH, Church TR, Flowers CR, Guerra CE, LaMonte SJ. Colorectal cancer screening for average-risk adults: 2018 guideline update from the American Cancer Society. Figure 4B. CA Cancer J Clin 2018;68:250–281.

Emphasizing the prevalence of CRC, the associated public health and economic burdens, and the significant preventive screening gap in the United States, ***Philip D. Parks MD, MPH*** *(Senior Director, Population Health, Exact Sciences)* shared the company's perspective on opportunities for CRC screening and associated population health strategies. Of particular concern, the burden of CRC within the group aged 45–49 years now exceeds that in the group aged 70–74 (Figure 3). In 2018, the American Cancer Society revised its CRC screening guidelines for individuals at average risk for CRC to include patients 45 years and older.

**FIG. 4.**
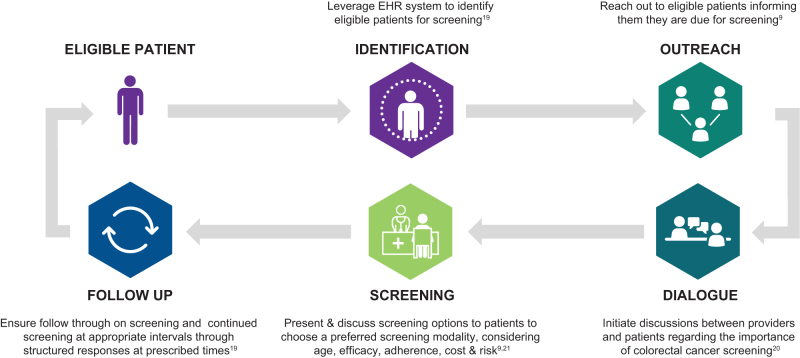
CRC screening process.^9,22–24^ CRC, colorectal cancer.

Low screening rates across different demographic and population segments can be attributed to reported patient- and system-level barriers. The evidence shows that early detection of CRC can lead to increased survival and decreased treatment costs. The company takes a risk-based approach to CRC screening via supporting shared decision making through clinical pathways and protocols (Figure 4).

Dr. Parks reviewed evolving health care trends, noting the importance of aligning technologies and solutions with health care stakeholders, public health needs, and patient preferences and values. He posed several questions:

- How do we calculate and report total costs of CRC screening? For example, administrative, programmatic, and other nonclinical costs are factors in the total cost of a FIT test.
- Should return on investment models be designed to help employers understand the costs and consequences of increasing CRC screening rates among their employees? If so, what inputs and outputs are needed for financial models?

### Key discussion points

- Advisors concurred that the increasing incidence of CRC in the group aged 45–49 is an important signal for population health advocates and for the nation's employers as they consider whether to cover the cost of CRC screening for their employees. National experts now recommend that the relevant HEDIS quality measure be expanded to include ages 45–49. This also ties in with a value-based approach.
- A measure of upstream mortality prevention through screening and early detection is very important but not well known among employers and other stakeholders. We know that the costs associated with CRC are enormous, but they are not well understood or easily quantified. Chief financial officers are concerned about the total cost of care for employees; a convincing case can be made that the total cost of care will decrease as we do a better job of screening.
- Regarding barriers to screening, advisors suggested systematizing screening, offering choices for screening when appropriate, leveraging technology, and encouraging provider offices to allow staff to work at the top of their licensure.
- Patients perceive trusted clinicians as “honest brokers.” The evidence shows that completion of preventive screening tests is demonstrably greater when the patient has a positive relationship with the ordering physician.^25^
- The best test is the one that will get done. CRC screening will not be driven solely by consumer demand; the consumer must be prompted to act by the physician. Providers can be incentivized via Medicare Advantage Stars credit and payment.

## Implementation of CRC Screening Programs: State of the Art and Science

Advisory Board member ***Ron Myers, DSW, PhD*** shared information on 3 “outreach” randomized controlled trials (RCTs) in CRC screening. The studies evaluated the impact of evidence-based standard interventions with and without tailored navigation in CRC screening for (1) a general population, (2) an African American population, and (3) a Hispanic American population. Researchers concluded that: (1) giving primary care patients a choice matters; (2) a centralized decision support and navigation outreach strategy can increase health system CRC screening and reduce disparities; and (3) infrastructure and protocols are needed to support implementation of an intervention in diverse populations.

Dr. Myers emphasized the importance of a collaborative approach and of developing a “learning community” concept. Health systems should employ cadres of navigators and provide training programs. State of the art navigation and decision support research is focused on empowering patients without adding to the primary care provider's burden.

### Key discussion point

Problems arise when a choice must be made among too many options; whenever possible, offer patients a choice between 2 options. Always give consumers the opportunity to choose the least invasive test.

## One Payer's Perspective: Cigna National Colon Cancer Screening Program

Advisory Board member ***Rajesh Davda, MD, MBA,*** presented an overview of Cigna's National Colon Cancer Screening Program. The main program features include 3 outreach modalities (ie, direct mail of FIT collection kit, brochure, web-based promotion), customization/collaboration (eg, coaches, case managers, Cigna Collaborative Care practices craft message from physician), and impact measurement via HEDIS data collection and reporting. Studies show that direct mailing and outreach resulted in statistically significant results compared with the control group.

An estimate of *avoided* medical costs for patients who tested positive and followed through with diagnosis and treatment is 4 times the cost associated with initial screening and colonoscopy.

An American Cancer Society/Cigna survey concluded that patients who completed a test kit were more likely to complete a test kit in the future.

## Preventing Colon Cancer Deaths and Disabilities: Value to the Provider, Payer, and Employer

Advisory Board member ***Raymond Fabius, MD,*** discussed the value of preventing CRC deaths and disabilities from the perspectives of 6 specific constituencies with different needs and expectations: (1) patients/health care consumers, (2) clinicians, (3) payers (insurance companies, health plans, third-party administrators), (4) purchasers (employers, government), (5) consultants (brokers), and (6) suppliers (pharmaceutical, laboratory, and disposable medical equipment).

When one of these constituencies has an issue, opportunity lies in helping to match that constituency with the best-suited service. For example: for providers, make things easier by helping to track quality scores such as CRC screening rates in their practices; for purchasers, help improve employee engagement with evidence-based guidelines to maximize productivity while demonstrating that the company cares about employee health and well-being.

Suppliers are an excellent source of knowledge and insights as well as potential distribution channels. For example, consider that >80% of people in the United States live within 8 miles of a supplier such as CVS or Walgreens.

Building a national culture of health starts with prevention strategies: primordial prevention (eg, clean water), primary prevention (eg, immunizations), secondary prevention (eg, screenings), tertiary prevention (eg, compliance with care management guidelines). We should strive to make it culturally unacceptable for a person or population not to be screened for CRC.

Where do we start?

- At the *national level*, CRC screening rates are suboptimal even for the best-performing states. Find ways to move the needle.
- At the *health plan level*, look at those who are doing the best job, find out why, and determine how to get others to improve.
- At the *employer level*, analyze cost data (eg, screening, high-cost claimants, long-term disability). There is a direct correlation between the health status of employees and the “culture of health” at an employer site. Conduct a pilot project at the best-performing employer site to establish a new benchmark in CRC screening and to show what is possible to achieve.
- At the *patient level*, create personalized incentives (eg, lower or zero co-pay for completing the screening process, financial rewards).

Suggested solutions Include: collaborative screenings (eg, universal cancer screening that meets employer, health plan, provider, and patient needs); education, marketing, and motivation; and creating competition around rewards and recognition (eg, best employer, best delivery system).

## Roundtable Discussion 1

Discussion questions were posted on the walls of the conference room and Advisors were asked to offer brief responses via Post-It Notes. A roundtable discussion expanded on these responses.

### How can Exact Sciences make a greater impact in helping health care stakeholders meet the NCCRT goal of 80% of eligible persons screened for CRC in every community?

Advisors agreed that patient check-in is the single most important element to ensure that information related to screening status is captured. Processes adopted by risk-bearing groups (accountable care organizations [ACOs], Medicare, capitated health plans) include filling out forms (including screening) when the patient arrives for an office visit, and figuring out how to incorporate this in the current workflow between the health care provider's office and the patient.

Advisors also concurred that there is a “disconnect” between the provider dialogue with the patient and the screening. They proposed pre-staging mt-sDNA in physician offices. To allow the patient a choice, FIT and mt-sDNA tests could be stocked and given directly to patients. Stocked kits must include bar codes that can be associated with specific patients. A designated staff member could be trained to do motivational interviewing.

Other recommendations included:

- Develop/implement a patient assistance program.
- Engage employer support networks. Noting that the average patient must take time off from work for colonoscopy may resonate with employers.
- Explore partnering opportunities with the Veterans Administration and Department of Defense.
- Work on developing studies to establish benchmarks.
- Explore ways in which the test might become the default test in the “Tele-doc” and health promotion space for consumers who do not visit doctors.

### How can value be demonstrated to payers, providers, and employers through population health strategies and initiatives?

Advisors encouraged the company to help establish a US benchmark for CRC screening. “Keeping well people well” requires a population health strategy that includes communicating with people and sharing anecdotes about how to avoid CRC.

Demonstrate value to providers and payers by emphasizing the test's contribution to a potential reduction in over-screening and overdiagnosis as well as under-screening and missed early diagnosis. In Medicare Advantage insurance markets, there is already potential for providing financial incentives to providers who improve and maintain Star ratings that are aligned with quality measures (including CRC screening rates). For employers, provide tool kits (videos, flyers) that are customized for their employee population(s).

From a quality perspective, systematic follow-up of positive tests is imperative. A positive mt-sDNA test is comparable to a suspicious mammogram, and all positive stool-based test results (including FIT, FOBT and mt-sDNA) should be followed by colonoscopy. Recruit teams and establish processes and protocols to ensure appropriate outreach and navigation for individuals with positive stool-based test results.

### How can value be demonstrated to payers, providers, and employers in a value-based payment paradigm?

*Purchaser/Employer perspective*: The corporate physician executive – as an “honest broker” – may be the driver for a mass audience. Explore the potential purchasing power of employer coalitions or onsite medical vendors to negotiate direct contract pricing for mt-sDNA.

Explain the value of early detection in terms of decreased overall cost, improved productivity, decreased short- and long-term disability, and quality-adjusted life years.

*Provider perspective*: For the provider, value lies in mastery, autonomy, rewards, and recognitions. Understand the mission of ACOs and equity-backed primary care providers and explore potential relationships.

*Health plan perspective*: Value lies in customer loyalty, member satisfaction, health outcomes, market share, and decreased total costs.

Consider whether patients on a 10-year negative colonoscopy screening regimen might elect to have mt-sDNA for their next screen rather than repeat colonoscopy.

### How can technology be leveraged to engage consumers on their health journey?

- Consider what happens in the case of a positive result for Medicaid beneficiaries and those who are uninsured. Is there financial assistance for these patients?
- Leverage technology to simultaneously reach/educate patients and incentivize providers (eg, in physician offices, a patient information intake system [eg, Phreesia] can replace the patient clipboard with a wireless touch screen and swipe card enabled PhreesiaPad).
- Understand employer data and methods of communication for engaging, educating, and empowering employees.
- Develop a personalized multimedia campaign to drive screening adherence using text messaging, social media, testimonials, and behavioral economics.

For guidelines to change, the case must be made at the societal level. If the data support societal change, advisors suggested that the company undertake a 10-year campaign with the goal of mt-sDNA becoming “the” first-line choice.

## Roundtable Discussion 2

### Comparisons Among Available CRC Screening Tests

*Comparative test performance and accuracy*: Accuracy is comprised of sensitivity and specificity. The clinical goal of a screening test is to balance potential risk and harm with high sensitivity and specificity. In the case of a CRC screening test, a highly sensitive test results in the lowest number of missed cancers; a highly specific test results in lowest number of nonproductive colonoscopies. The sensitivity of FIT is 74% versus mt-sDNA at 92%; the specificity is 95% and 87%, respectively. Over a 3-year period of annual FIT tests vs. 1 mt-sDNA test, the specificities are similar.^26^ The diagnostic accuracy of colonoscopy is dependent on factors such as the right prep, the right skill set, and the right facility. If mt-sDNA is a more accurate test than FIT, the company should generate evidence and compete for preferred screening test status among payers, employers, and other purchasers.

*Accountability*: Today, accountability for any screening test rests with the primary care provider. However, some of the specialist's “bread and butter” volume comes from doing things that are more appropriately done by primary care providers (eg, managing prostatitis). The world is moving toward capitated screening, and the process is best managed by primary care physicians.

*Culture change*: Colonoscopy is considered “gospel” and the “gold standard” in primary care today, and health plans will be followers rather than leaders with respect to clinical practice recommendations. Managed care plans might consider partnering with the company; however, there is low probability of early adoption without real-world clinical evidence of the value of mt-sDNA in an appropriate population.

Mt-sDNA is a screening test for detecting adenoma and CRC whereas colonoscopy is a procedure to detect adenoma. Perhaps the primary care physician should be held accountable only for screening tests; accountability for diagnostic colonoscopy would rest with the gastrointestinal specialist.

Delivery systems can contribute but will not lead cultural change. Early adoption happens within benchmark organizations where employees are encouraged to take care of themselves *and* their coworkers. Cultural transformation makes it socially unacceptable to not have preventive screening. Lifestyles have overwhelming influence on cancer rates. Promotion of behaviors such as weight management, healthy eating, nonsmoking, and regular exercise must be encouraged.

*Budget impact/cost effect*: If a screening test is part of a bundle, it has potential to move to the front line. The best result will occur when payers and providers are aligned. Most employers will not purchase individual point solutions and will rely on health plans to administer benefits and health care resources.

*Interventions*: The behavior modification industry has a wide variety of software and patient connectivity tools and payers have data. Avoid reporting fatigue (ie, multiple doctors asking about the last colonoscopy) by centralizing the population-level data for CRC screening. Use technology to identify eligible patients. Target patients with incentives based on individual needs (eg, gift cards and/or free meals based on spending patterns and needs).

Develop pilots to test interventions (eg, incentives, provider offices).

What has worked?

*Engaging consumers* – JCPH helped build the CVS Minute Clinic model and Philadelphia is home to the Convenient Care Association. Patients could pick up test kits at Minute Clinics. The complete process can be done at Walgreens. Primary care practitioners are on-site at CVS/Aetna, Walgreens/Humana.

*Personalization* – Studies show that it is important to determine how to deliver reminders and send material with photos of and references to someone who is similar to the intended recipient.

## Roundtable Discussion 3

Dr. Nash asked Advisors for their 2 most important takeaway messages; their responses are summarized below:

### General observations and recommendations

Clinicians agreed that a screening test should be readily accessible (ie, inexpensive, physician prescription unnecessary, “order on Amazon”). By that definition, colonoscopy is not a screening test. Mt-sDNA is a tool that can help bring preventive screening back into the primary care setting; the test might even be done during an office visit.

Advisors also suggested that incorporating the very “human” aspects of CRC (eg, impact on patients, caregivers) can be helpful in personalizing communications. Stories related to early detection and survival can put a real “face” on CRC.

### Re: employers

Help employers understand the total costs of CRC for their unique workforces by developing information on the incidence of CRC and its associated costs in the context of overall health care (including oncology) spending and indirect costs (eg, disability, labor replacement). Design a study to answer population-based questions. For example, integrated delivery systems can use CRC screening data and absentee data from their employee populations to calculate cost savings over a 10-year period.

In general, an employer's workforce can be sorted into 4 distinct groups in terms of health care seeking behavior: (1) 20% who research and access care independently (eg, engineers), (2) 30% who prefer shared decision making with a trusted clinician, (3) 30% who want their doctors to make all decisions for them, and (4) 20% who are completely divorced from the health care system (eg, young men). Each cohort requires a different approach.

Other valuable advice included:

- Learn to speak the language of employers and engage in conversations with employer coalitions.
- Engage with data analytics groups to show value to employers across the board.

### Re: providers

Consider what it would take to screen 100% of the population according to clinical guidelines and take action. For instance:

- Design a “one-click solution” and help train staff to work with patients in a standardized way.
- Promote benchmarks in each constituency as well as global benchmarks, and do not underestimate the power of “shaming”; providers are very competitive.

Explore relationships with ACOs and equity-backed primary care practices.

### Re: research

Plan and execute demonstration projects, including: (1) deciding what to put in place with an employer/purchaser or health system, (2) bringing stakeholders into alignment on goals and objectives, and (3) implementing evidence-based interventions to drive the initiative and create real-world examples of improving screening rates.

Address disparities. In addition to rural populations, consider the 25%-40% of commercially insured consumers who have no medical home^21^ and develop a plan for screening the medically homeless.

### Re: payers

Collaborate on bundling arrangements for large GI practices to manage the screening and diagnostic components of testing. Screen with the most appropriate test (in terms of combined efficiency, cost, patient-centeredness, staged diagnosis) and determine the associated metrics.

## Conclusion

JCPH, in collaboration with Exact Sciences, organized and convened an advisory meeting of health professionals to discuss the value proposition of the mt-sDNA test for CRC screening in terms of clinical quality, risk management, finances, and population management. Expert opinions were aligned regarding the profound negative implications and substantial clinical, financial, individual, and population health costs associated with a failure to meet the NCCRT goal of 80% CRC screening rate in every US community. Likewise, there was concurrence among experts that CRC screening options for individuals at average risk for CRC must be effective, accessible, easy to use, and offered as part of a shared (clinician/patient) decision-making process that takes into account disparities among cultural and ethnic groups in urban and rural settings. The thoughtful discussions among Advisors revealed multiple opportunities for immediate implementation as well as recommendations for further review and exploration.
